# Why Animal Experiment Is Necessary

**Published:** 1921-05-07

**Authors:** 


### RESEARCH DEFENCE.

### Why Animal Experiment is Necessary.

We are at present living in the age of propaganda, and the argument for propaganda rests on a well-founded conviction of the necessity of educating public opinion. Nothing really effectual can now be done unless the public are fully convinced of the advantages to be gained by following a certain course, and this applies as much to laws which are enacted as to procedures which benefit health, and depend for fulfilment upon voluntary acquiescence. Before instituting health regulations we must convince public opinion of their necessity.

### "Vivisection" in Practice.

Now these remarks apply with special force to the subject of this article, and we shall therefore endeavour to support our opinion by a brief reference to some of the benefits to mankind of experiments on animals. In the first place we would like to remark that we have been flooded for many years with literature against experiments on animals, and that practically all the arguments contained in the many pamphlets are founded on the catchword "vivisection." This literally means cutting up animals while alive and *fully conscious*, regardless of the pain inflicted, and to see that this interpretation is intended we need only cite the exhibition on posters of the crude and gruesome pictures representing animals being tortured with their eyes wide open and exhibiting all the evidences of acute mental and physical anguish. In reply to this we can confidently assert that no experiment involving a more serious procedure than simple inoculation is ever done except under deep anæsthesia, and with as little physical or mental suffering as is experienced by a human being under similar circumstance. Also let us remember that animals do not suffer the anticipatory mental agony before an operation that many patients pass through. Further, they are not allowed to endure the pain and suffering which accompany recovery from the anæsthetic, since, if they are likely to suffer they are killed before coming out of the anæsthesia.

### The Benefit to Medicine.

In the next place the benefits accruing to experimental research are so numerous that it would be impossible in a short paper to enumerate them all, but it will lead to a clearer conception of these if we consider them briefly under the following heads:

1. Physiological. 2. Pathological. 3. Diagnostic. 4. Therapeutic. 5. Medico-legal.

1. *Physiological.*—Under this head we may mention all those experiments on the heart and circulation, by means of which we have enriched our knowledge of the action of the heart and vessels in health, and without which we could never have understood their functions. Also note the numerous experiments on digestion by men like Pawlow, by means of which we have gained an insight to the processes of digestion in the stomach and bowels, and incidentally gained a knowledge of the functions of the liver and other important internal glands. Under this head we may also include experiments on the brain, by means of which we can localise its function, the knowledge of which enables us to locate cerebral tumour, missiles, etc., and effect their removal.

2. *Pathological.*—Under this heading we can include those experiments of inoculation and feeding with a view to the artificial production of cancer, and attempts to find out its cause; also the feeding experiments which have produced rickets—a most serious and disfiguring disorder of childhood. The causes of the large class of diseases known as infectious can only be investigated by their experimental production in animals.

3. *Diagnostic.*—Many diseases—notably tuberculosis—of obscure beginning and affecting such internal organs as the kidneys cannot be diagnosed in the early and curable stages without experimental inoculation of guinea-pigs, and a variety of other diseases could be named where similar procedure is necessary.

4. *Therapeutic.*—Some of our most brilliant therapeutic triumphs are due to experiments on animals, and one only need be mentioned—in the production of diphtheritic antitoxin from the serum of the horse.

5. *Medico-legal.*—This is the last example. In the detection of obscure ptomaine poison from foods, or of those insidious poisons such as curare, which may be administered with criminal intent, feeding and inoculation experiments on animals are the only means by which their presence can be detected.

From this brief summary it must be evident that modern advances in medicine are based largely if not entirely on experiments in animals, and, as we mentioned last week, we welcome the decision to publish the journal of Research Defence again, and are more than gratified that it will be made accessible to the general public.

